# Reports from the Laboratory of the Royal College of Physicians, Edinburgh

**Published:** 1891-06

**Authors:** 


*Reports from the Laboratory of the Royal College of Physicians, Edinburgh.* Edited by J. BATTY TUKE, M.D., and D. NÖEL PATON, M.D. Vol. III. Edinburgh: YOUNG J. PENTLAND. 1891.

This third volume of the *Reports* of the Edinburgh Laboratory fully keeps up the high reputation of the two preceding ones. The papers cover a good deal of ground, being on widely different subjects; so that there is matter here for all members of the profession, with whatever branch of it they may be especially concerned—whether with medicine, obstetrics, bacteriology, physiology, or morbid anatomy. Although investigations published under such auspices have naturally been conducted on pure scientific lines, and the reports of them bear everywhere the stamp of this origin, it must not be concluded that they have no practical everyday bearings; on the contrary, one of the most striking points about the book is the close relation that the experiments, of which the account is here given, maintain with the details of clinical practice, and the real aid that can be gained from a study of them towards carrying this out on rational grounds. The observations are obviously the outcome of much and long-continued labour, and the conclusions obtained have been closely scrutinised and duly weighed.

The first article is a most able and elaborate account of the pharmacology of morphine and its derivatives, by Mr. Dott and Dr. Stockman. Next follows a short report by Dr. Stockman on the excretion of balsams in the urine, in which he shows that the four balsams of the Pharmacopœia can be given in as large a dose as is ever desirable in practice, without fear of inducing nephritis or albuminuria. He thinks that in some at least of the recorded cases of albuminuria after administration of balsams a resinous body in the urine has been mistaken for albumen.

Dr. Helme gives an interesting study of the physiology of the uterus, and the action of drugs upon it. His experiments show that contraction of the uterus is coincident with diminution of the blood-flow through it, contrary to what is observed in striped muscle, and that the rhythmical character of the uterine contractions can always be traced. He gives full directions, based on experiment, for the employment of the hot and cold uterine douche, and discusses fully the action of ergot, chloral, and camphor.

Then come two papers by Dr. Lovell Gulland: the first, on the origin, nature, and variety of leucocytes, in which the different theories are critically discussed, and the author's own observations on the points at issue given; the second, on the development of adenoid tissue, with especial reference to the tonsil and thymus. This paper and the one following, by Dr. Chas. Kennedy, on a case of cystic disease of the liver and kidneys, are illustrated by good drawings of microscopic sections.

Dr. Nöel Paton and Dr. Balfour contribute an important paper on the composition, flow, and physiological action of the bile in man, from a series of observations in a case of complete biliary fistula. They are led to the general conclusion that the bile is rather an excretion than a secretion which performs an essential part of the digestive process, and that the entrance of the bile into the intestine is not necessary for the maintenance of health. In its absence, the only digestive disturbance produced is the diminished absorption of fats, and even of these 70 per cent. are still absorbed. They think that the prevalent ideas as to the quantity of bile excreted and of solids produced are too large. Of the drugs that increased the flow of bile, calomel and salicylate of soda appeared to be the most active, and ox-gall in less degree. The clinical use of calomel would seem, therefore, to be justified by their observations. Dr. Nöel Paton also has a paper on the influence of muscular work on proteid metabolism, in which he points out the inaccuracy of the conclusions arrived at by Argutinsky and Krummacher.

An article by Dr. J. C. Webster on the nerve-endings in the labia minora and clitoris, with especial reference to the pathology and treatment of pruritus vulvæ, in which he says that the removal of the affected parts is the only means of complete cure, is followed by an interesting contribution by Dr. Berry Hart on the anatomy and mechanism of early abortion. A description of an improved method of preparing large sections of tissues for microscopic examination is given by Dr. Webster.

A paper by Drs. Woodhead and Cartwright Wood on bacterio-therapeutics, in which the theory of immunity is discussed in a clear and scholarly manner, will be read with interest and profit at the present time, when attention is so strongly directed to this subject.

The last research is one by Drs. Cartwright Wood and Maxwell Ross on the influence which the process of inflammation exerts on the course of infection as exemplified in anthrax and erysipelas. As an outcome of their observations, they give a method of treatment for the latter affection which seems likely to be of service in cutting short the spread of the disease.

It is impossible in the course of a short notice to give more than a mere outline of the contents of the book, but the above remarks will indicate the extent and the scientific and practical interest of the researches described in it. To most of the papers is appended a bibliography, and many of them are accompanied by charts and drawings which add to their value. We must congratulate all concerned in the production of this work, and we shall look forward to succeeding volumes from the same source. The general appearance and style of the book in printing, binding, etc., is excellent.

